# The Microbiome in the Development and Treatment of Inflammatory Bowel Disease

**DOI:** 10.3390/biomedicines14081754

**Published:** 2026-08-04

**Authors:** Sanzhar Zhetkenev, Roman Konovalov, Azamat Akhmetkaliyev, Eva Sonnenberg-Riethmacher

**Affiliations:** Department of Biomedical Sciences, School of Medicine, Nazarbayev University, Astana 010000, Kazakhstan

**Keywords:** probiotics, microbiome-based therapeutics, inflammatory bowel disease, Crohn’s disease, ulcerative colitis, microbiota, genetically engineered bacteria, dysbiosis

## Abstract

Inflammatory bowel disease (IBD) is a chronic inflammatory disorder of the gastrointestinal tract that arises from a complex interplay of genetic susceptibility, immune dysregulation, environmental exposures, and altered host–microbiome interactions. Increasing evidence identifies the gut microbiota as a central component of IBD pathogenesis. In healthy individuals, the intestinal microbiota supports epithelial integrity, metabolic homeostasis, immune education, colonization resistance, and bidirectional gut–brain communication. In IBD, this ecosystem is disrupted by reduced microbial diversity, expansion of pathobionts, and broader functional alterations affecting community stability and metabolic output. Importantly, these changes are increasingly viewed not merely as consequences of inflammation, but as active contributors to disease development and persistence. Dysbiosis may also influence neuroimmune signaling through the gut–brain axis, linking microbial metabolites, intestinal barrier dysfunction, enteric nervous system activity, and psychological comorbidities frequently observed in patients with IBD. This review provides a comprehensive overview of the role of the gut microbiota in IBD, beginning with its physiological functions in intestinal homeostasis and the evidence linking dysbiosis to disease pathogenesis, followed by a critical evaluation of current microbiome-based therapeutic strategies, their translational challenges, and prospects for personalized microbiota-directed interventions. Approaches such as fecal microbiota transplantation (FMT), probiotics, live biotherapeutic products, and genetically engineered bacteria aim to restore microbial balance and modulate intestinal inflammation. Among these, FMT has provided the strongest proof-of-concept for microbiome restoration, whereas probiotic efficacy remains variable and strain-dependent. Emerging defined microbial consortia and engineered bacterial platforms offer improved standardization and mechanistic precision, but their clinical application remains limited by challenges related to engraftment, durability of response, safety, and treatment optimization. Collectively, current evidence supports gut microbiota as both a key determinant of IBD pathogenesis and a promising therapeutic target, underscoring the need for more precise and personalized microbiota-directed approaches in IBD management.

## 1. Introduction

IBD is a chronic, multifactorial inflammatory disorder of the gastrointestinal tract. It includes two main forms, Crohn’s disease (CD) and ulcerative colitis (UC). A third, less common form is IBD unclassified (IBDU), which is used when the diagnostic criteria for the main forms do not meet owing to overlapping or indeterminate features [1,2,3]. In terms of disease phenotype, CD can affect any part of the gastrointestinal tract and is typically characterized by transmural, discontinuous inflammation [4,5], while UC is usually limited to the colonic mucosa and extends continuously proximally from the rectum [6,7]. IBDU, in turn, can share both features of CD and UC [3,8].

Although IBD was long viewed as a disease of the Western world [9,10], its epidemiology has shifted dramatically, with incidence rising rapidly in industrialized countries [11,12,13]. Interestingly, the rate of new cases has plateaued or even decreased in developed countries while prevalence still remains high [13]. This divergence between emerging and developed regions, decoupled from genetic background, strongly implicates environmental and lifestyle factors as important factors of disease pathogenesis [14,15,16,17,18]. These factors point to a broader pathogenic interplay in IBD involving genetic susceptibility [19], immune dysregulation [20], environmental exposures [21,22], and altered host–microbe interactions [19,23].

Genome-wide association studies have recognized many loci to be associated with IBD, many of which are involved in microbial sensing, autophagy, barrier maintenance, cytokine signaling, and immune response [24,25,26]. However, inherited susceptibility alone cannot fully explain disease etiology or the increasing global burden of IBD. Rather, disease development seems to be linked to a deregulation of intestinal homeostasis in genetically predisposed individuals exposed to specific environmental stimuli [27]. In this context, altered host–microbe interaction is emerging as an important mechanistic link between environmental risk factors and chronic intestinal inflammation [28,29].

The intestinal microorganisms form a dynamic ecosystem referred to as microbiota. In healthy individuals, symbiotic host–microbe relationships maintain microbial homeostasis and support essential physiological processes [30,31]. In IBD, this balanced ecosystem is impaired, leading to dysbiosis [32,33,34,35]. Importantly, dysbiosis is no longer considered simply a secondary consequence of inflammation. Instead, accumulating evidence suggests that changes within the microbial community may actively contribute to disease pathogenesis [35,36,37,38,39].

This bidirectional relationship between the host and the microbiota has generated increasing interest in microbiota-targeted therapeutic strategies. Diet-based strategies, prebiotics, probiotics, synbiotics, antibiotics, and fecal microbiota transplantation (FMT) are among the approaches being investigated as adjuncts or alternatives to conventional IBD therapy [40,41,42]. Yet, clinical efficacy remains variable, reflecting differences in disease subtype, disease location, microbial composition, host genetics, and treatment context [43,44,45]. At the same time, established pharmacological therapies remain limited by adverse effects, primary non-response, secondary loss of response, and incomplete mucosal healing [46,47]. Accordingly, understanding the role of the gut microbiota in the development and treatment of IBD may provide important insights into disease mechanisms and help guide more targeted and personalized therapeutic approaches. This review provides an overview of the role of the gut microbiota in IBD pathogenesis and critically assesses current microbiota-based therapies, translational challenges, and future personalized treatment strategies.

## 2. The Gut Microbiota in Health and IBD

### 2.1. Gut Microbiota in Health

The human gastrointestinal tract harbors trillions of microorganisms of highly diverse species comprising viruses, bacteria, protists, and fungi [32,33,34,48]. This ecosystem is relatively compositionally stable, with bacterial components dominated by the representatives of phyla Firmicutes (Bacillota) and Bacteroidetes (Bacteroidota), together with less abundant populations of Actinobacteria, Proteobacteria, Verrucomicrobia, and Fusobacteria [49]. Notably, the gut microorganisms are not uniform along the gastrointestinal tract but vary substantially between anatomical regions [50,51,52]. Each segment has its own unique microbial signature shaped by pH, oxygen availability, nutrient content, and transit time of the microorganisms within anatomical niches [53,54,55]. Furthermore, the microbiota exhibits spatial organization within each intestinal segment, with differences between luminal and mucosal surfaces [52,56,57,58,59]. For example, mucosa-associated microbiota (MAM) are in closer contact with epithelial and immune cells and therefore seem to play a more prominent role in shaping host immune responses [60].

Functionally, the gut microbiota ensures the homeostasis of the digestive system. Commensal microorganisms are involved in the digestion of complex polysaccharides [61], production of vitamins [62,63,64] and antimicrobial compounds [65,66,67], synthesis of short-chain fatty acids (SCFAs) [68,69,70,71], which serve as an essential energy source for colonocytes [72]. Moreover, SCFAs support mucosal integrity by promoting epithelial-cell metabolism and tight-junction maintenance, thereby limiting intestinal permeability and bacterial translocation [73,74]. SCFAs, namely butyrate, also modulate mucosal immunity through the differentiation of functional colonic regulatory T cells by increasing histone H3 acetylation at the *Foxp3* locus, thereby enhancing *Foxp3* expression and ameliorating T-cell-mediated experimental colitis in mice [75]. Another study also highlights the importance of SCFAs. In a murine T-cell-transfer colitis model, butyrate attenuated intestinal inflammation by suppressing Th17-cell differentiation and promoting IL-10 production in microbiota antigen-specific CD4-positive T cells through histone deacetylase inhibition, independently of GPR43 signaling [76]. Consequently, reduced abundance of SCFA-producing microorganisms in IBD may contribute to impaired barrier function and persistent mucosal inflammation.

The microbiota also supports colonization resistance by competing with pathogens for nutrients and niche occupation, thereby maintaining an environment unfavorable to pathogen overgrowth [77,78,79]. Collectively, these processes facilitate epithelial integrity [35,36], metabolic regulation [37,38,39], immune modulation [41,42,43], and colonization resistance against pathogens [80,81]. Overall, a healthy gut microbiota is represented by a variety of microorganisms that are stably integrated into a multifunctional ecosystem contributing to intestinal homeostasis.

### 2.2. Dysbiosis in IBD

**Figure 1:**
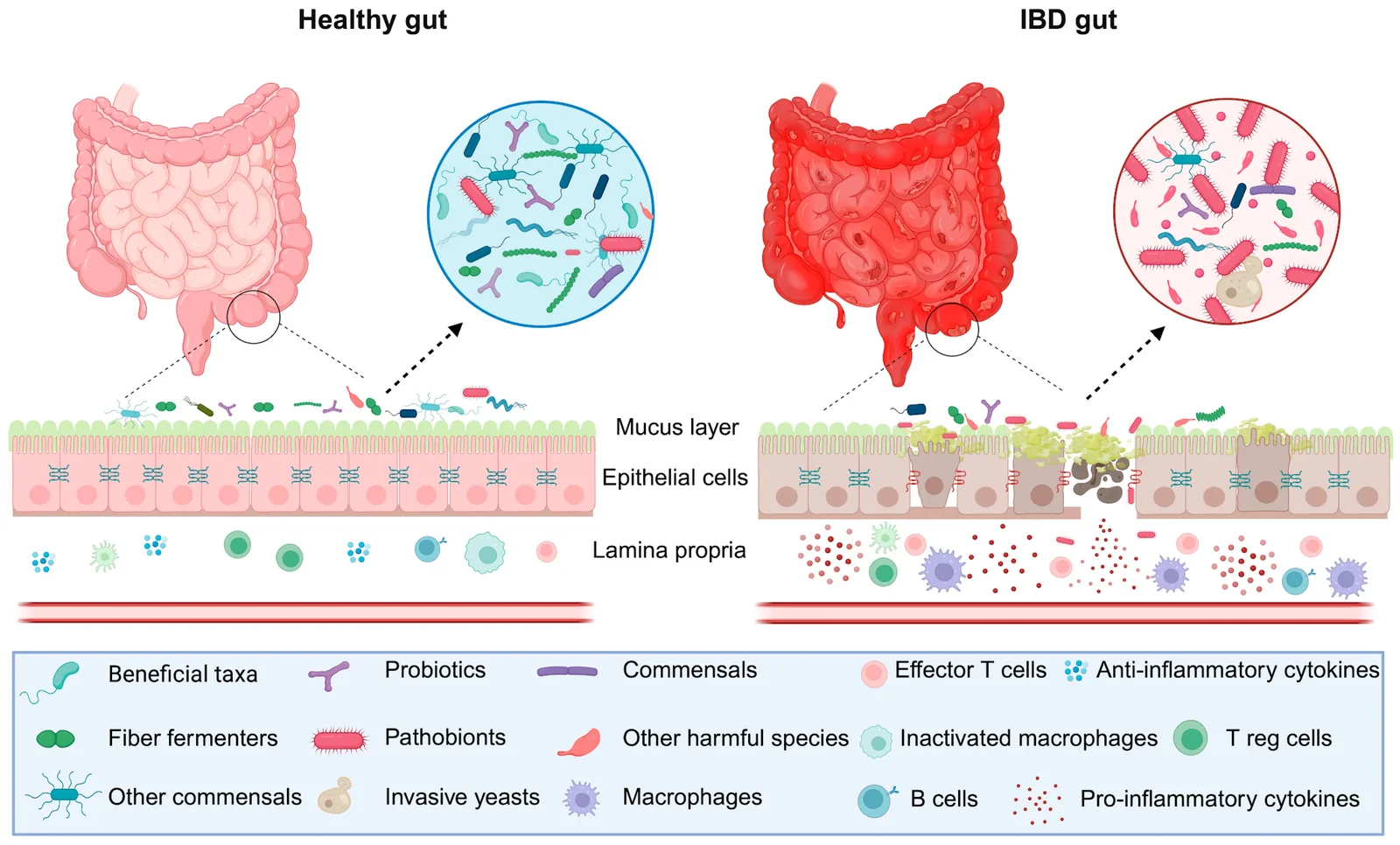
Composition of gut microbiota in health and IBD. A healthy gut is defined by its diversity and balance, the intact mucus and epithelial barrier, and immune homeostasis that maintain normal intestinal function (**left panel**). Conversely, gut microbiota dysbiosis in IBD patients is characterized by reduced microbial diversity; loss or substitution of microbial beneficial taxa, such as *Ruminococcaceae* and *Leuconostocaceae*, that are abundant in healthy guts; enrichment of pathobionts, which gained pathogenicity under IBD-related conditions and other harmful species (e.g., AIEC, *Ruminococcus gnavus*, *Atopobium parvulum*); expansion of invasive yeast species, such as *Debaryomyces hansenii*, *Candina albicans*, and *Malassezia restricta*; and a disturbed epithelial barrier (**right panel**). Created in BioRender. Riethmacher, E. (2026) https://BioRender.com/8nmwdub (accessed on 10 July 2026).

A disruption of the balance within the microbial community is termed dysbiosis and is considered a hallmark of IBD [82,83,84,85,86]. This dysbiotic state is characterized by alterations in composition, structure, and function of gut microbiota (Figure 1). While specific compositional alterations will be discussed in the next chapter, IBD-associated dysbiosis is also characterized by an altered network topology and reduced ecosystem stability. Recent work has shown that dysbiosis is accompanied by reduced modularity and disrupted community structure, suggesting a less stable and less organized ecosystem [87]. Consistent with this, a network-based analysis of the IBD cohort demonstrated that affected patients exhibit higher clustering coefficients and markedly lower modularity compared to healthy controls, supporting increased co-dependence among taxa, loss of community, and a more consolidated but less organized microbial network [88].

At the functional level, metagenomic studies have shown that the IBD microbiome is enriched in genes involved in oxidative stress responses, nutrient transport, and altered metabolic pathways, reflecting functional adaptation to the inflamed intestinal environment [89,90]. Collectively, these changes within the microbiome are likely to influence disease pathogenesis, contributing to intestinal inflammation.

From a clinical perspective, alterations in the IBD microbiome are increasingly recognized as an important contributor to disease pathogenesis. In UC, distinct microbial community states were associated with disease severity, and the most severe state showed the highest simple clinical colitis activity (SCCA) [91]. Similarly, another study indicates that the pediatric IBD microbiota profile shows a gradient where greater dysbiosis is associated with higher calprotectin levels [92]. Importantly, patients exhibiting clinical remission frequently continue to show signs of dysbiosis [86,93], suggesting that these microbiome changes are not merely a consequence of overt symptoms but may reflect an underlying disease-associated state. In agreement with this, healthy family members of patients with CD demonstrate altered microbiota composition and subclinical inflammation of the intestine, making them more prone to develop the disease [94]. This again could point toward the intricate relationship between genetic predisposition and microbiome composition.

In summary, IBD-associated dysbiosis alters network topology, reduces ecosystem stability, and leads to functional adaptations to an IBD-promoting environment. Collectively, these pieces of evidence establish dysbiosis as a contributor and potential biomarker of IBD, highlighting the rationale for microbiome-targeted therapeutic strategies.

### 2.3. Taxonomic and Functional Alterations in the IBD Microbiome

The most consistent findings across the studies for IBD-associated dysbiosis are reduction in microbial diversity, depletion of species abundant in the normal healthy state, activation of pathobionts, and expansion of potentially pathogenic organisms. Multiple cohort studies and systematic analyses have demonstrated that IBD patients exhibit significantly lower diversity, with this reduction being more pronounced in CD than in UC [95,96,97]. This loss of diversity correlates with disease activity, inflammation markers, and clinical features [96,98]. Reduced diversity likely reflects a simplified microbial ecosystem with limited functional outcome. Critically, low alpha-diversity persists even in patients in clinical remission [93], suggesting that dysbiosis is not solely a consequence of active inflammation but may represent a fundamental feature of IBD pathogenesis.

Together with loss of diversity, IBD is characterized by consistent shifts in microbial composition. IBD patients demonstrate a reduction in species associated with a healthy microbiota, including representatives of families *Ruminococcaceae*, *Leuconostocaceae* and the genera *Bifidobacterium* and *Lactobacilli* [33,86,99]. In particular, UC patients exhibited decreased levels of anti-inflammatory, butyrate-producing bacterial species from the Firmicutes (Bacillota) phylum, specifically *Roseburia hominis* and *Faecalibacterium prausnitzii* [100]. Similarly, the reduction in *Faecalibacterium prausnitzii* has also been reported in CD [101]. A recent study also found a correlation between the reduction in certain Bacteroidetes (Bacteroidota) species and UC activity [102]. Together, the depletion of these taxa results in reduced SCFA production and weakened mucosal immune regulation.

In contrast to the loss of non-pathogenic microorganisms, the population of pathobionts, commensal microbes that become harmful under specific conditions, tends to increase during IBD [103]. One of the best-studied microbes is the adherent-invasive *Escherichia coli* (AIEC) [104]. In vitro, AIEC strains recovered from lesions of CD patients invaded intestinal epithelial cells and persisted in murine macrophages, inducing TNF-α secretion without triggering cell death [105]. In a recent study using human macrophages, AIEC strains formed intracellular bacterial communities and persisted intracellularly and induced inflammatory responses, including TNF-α and IL-6 production [106]. Among other pathobionts, increased abundance of bacterial species such as *Ruminococcus gnavus* [107,108], *Atopobium parvulum* [109,110], and fungal species *Debaryomyces hansenii* [111,112], *Candida albicans* [112,113,114], and *Malassezia restricta* [112,115,116] were consistently linked to IBD. The proposed mechanisms by which these pathobionts contribute to IBD largely converge on common pathways, including induction of pro-inflammatory cytokines [105,106,113,115], disruption of mucosal integrity [111], and potentially affecting mitochondrial function [110]. Moreover, some pathobionts were shown to be involved in epithelial invasion, biofilm formation, and production of harmful metabolites [117,118,119].

Current evidence shows that gut dysbiosis in IBD is not limited to alterations in bacterial and fungal microorganisms but also affects the virome and archaeome. The healthy gut virome consists mainly of bacteriophages [120]. Comprehensive analysis demonstrated IBD-associated imbalance within the viral population, with *Caudovirales* being increased and *Petitvirales* phages being decreased across the worldwide IBD population [121]. This imbalance in the bacteriophage community can potentially affect bacterial species via predation and/or horizontal gene transfer (HGT) [122], resulting in an increase in species that obtain disease-related pathogenicity and a reduction in abundant species under normal health conditions. Similarly, the archaeal community is also affected in IBD with pronounced reduction in methane-producing archaea such as *Methanobrevibacter smithii* [123]. This archaeal alteration could disturb fermentation processes and affect intestinal transit time, further contributing to disruption of gut ecosystem homeostasis [123,124].

Importantly, different IBD subtypes demonstrate distinct patterns in taxonomic profile. Teofani and colleagues (2022) showed that UC is linked to alterations in *Bifidobacteriaceae*, *Rikenellaceae*, *Christensenellaceae*, *Marinifilaceae*, *Desulfovibrionaceae*, *Lactobacillaceae*, *Streptococcaceae* and *Peptostreptococcaceae* families, whereas the CD signature includes changes within *Christensenellaceae*, *Marinifilaceae*, *Rikenellaceae*, *Ruminococcaceae*, *Barnesiellaceae* and *Coriobacteriaceae* families [125]. A more recent study also appreciated shared signatures between UC and CD but also observed that CD was characterized by an increased abundance of *Escherichia coli*, which was associated with antibiotic resistance determinants and virulence factors typical of AIEC [126]. Another study also supported the pathology-specific alterations in microbiota between IBD forms and also links it to specific metabolic alterations. To be precise, UC-associated microbiota are enriched in glycolytic, pyruvate, and glycan metabolism pathways, while CD-associated microbiota are linked to amino acid and simple carbohydrate metabolism [127]. These findings suggest that UC and CD not only differ taxonomically but also harbor distinct functional microbial landscapes, with implications for disease-specific therapeutic targeting. Altogether, alterations within the commensal microbial community disturb intestinal homeostasis, negatively affecting the overall balance of gut microbiota and playing a well-established role in IBD pathogenesis.

The gut microbiota composition is increasingly recognized as having collective functional potential and metabolic effects rather than simple presence or absence of specific taxa [128]. Shotgun metagenomics and strain-resolved studies that demonstrate functional features, in contrast to taxonomic identification, better capture the ecological roles of gut microbiota [129]. The human microbiome’s gene composition or functional capacity is highly preserved, even though its taxonomic composition differs greatly amongst individuals. This suggests an ecological characteristic known as functional redundancy [130]. Research reveals that butyrate synthase genes are dispersed by several distinct pathways and some horizontal gene transfer among phylogenetically dissimilar Firmicutes (Bacillota) lineages [131]. Overlapping metabolic pathways are encoded by numerous phylogenetically different taxa, illustrated in a study which have found predicted producers that yielded similar SCFAs within 36 families [132]. A study comparing species found in the healthy human gut, *Bacteroides vulgatus* and *Bacteroides distasonis*, and Bacteroides outside of the gut has found that the ability of gut-colonizing Bacteroidetes (Bacteroidota) to change their cell surface, perceive their surroundings, and gather nutrients from the distal intestine has been significantly impacted by lateral gene amplification, mobile elements, and gene transfer [133]. Another interesting finding was described in research on the Japanese population gut microbiome where CAZyme genes encoding enzymes unique to marine algal polysaccharides were transferred to *Bacteroides plebeius* from a seaweed saprotroph closely related to *Zobellia galactanivorans* via HGT [134]. Analysis of microbiome community models from an IBD study revealed that despite decreased species diversity in IBD subjects, functional redundancy increased for certain metabolites such as hydrogen sulphide, indicating the potential of such alterations to provide insights beyond species diversity alone [135]. According to a study that examined 9838 metagenomic samples from 29 different nations, varied taxonomic community architectures converge around three functional archetypes, demonstrating that different compositions can inhabit the same functional space. Despite sharing Archetype 3 associated with high nitrogen/amino-acid processing, IBD samples were distinctly enriched for NAD salvaging, amino-acid biosynthesis, an *E. coli*-specific fatty-acid pathway, and peptidoglycan biosynthesis in comparison to healthy controls [128]. Data obtained from the metagenomic fecal samples of European and US cohorts demonstrated that propionate production was driven by *E. coli* in CD but by *Anaerostipes hadrus* in UC and healthy controls [136]. This illustrates that the same function can be carried out by entirely different taxa depending on disease state. Taken together, these results indicate that functional profiling approaches that utilize metagenomic pathway analysis, metabolomics, or meta-transcriptomics should be used alongside taxonomic profiling to appropriately define IBD-associated dysbiosis and aid in therapeutic approaches that target dysregulated functions via multiple taxa or direct metabolites instead of just single taxa.

### 2.4. Gut–Brain Axis

The gut microbiota communicates bidirectionally with the central and enteric nervous systems via neural (vagal and spinal afferents), endocrine (neuroactive hormones and peptides), immune, and metabolic (microbial metabolites) pathways, collectively establishing the gut–brain axis [137,138]. In the context of IBD, this axis is potentially relevant because dysbiosis may modulate intestinal inflammation not only through local epithelial and immune mechanisms but also via altered neuroimmune signaling and enteric nervous system function [139,140]. SCFAs such as acetate, propionate, and butyrate are key mediators of gut–brain communication [141]. They are not only important for epithelial barrier integrity as discussed above but also act on neural and glial cells to affect neurotransmitter systems and neuroinflammation [142]. The depletion of SCFA-producing taxa frequently observed in IBD may therefore contribute to disease pathogenesis beyond impaired colonocyte nutrition and local immune regulation. To illustrate, alterations in SCFA availability may compromise enteric nervous system homeostasis, as SCFA supplementation restored enteric neuronal numbers and promoted enteric neurogenesis following antibiotic-induced microbiota depletion in mice [143]. Moreover, it has been shown that enteric glial cells can internalize and respond to SCFAs through intracellular signaling, while butyrate induces neurochemical plasticity of enteric neurons and modulates colonic motility [144,145]. Thus, altered SCFA availability in IBD may further perturb enteric nervous system homeostasis and contribute to altered gut–brain and neuroimmune signaling, which could be a potential link between microbial dysbiosis, intestinal inflammation, and extraintestinal manifestations of IBD.

Importantly, the gut–brain axis is a bidirectional phenomenon. Chronic intestinal inflammation and dysbiosis may influence central nervous system function through circulating inflammatory mediators, microbial products and metabolites, and vagal signaling [139]. Conversely, psychological stress and dysregulation of the hypothalamic–pituitary–adrenal axis have been implicated in changes to intestinal permeability, gastrointestinal motility, mucosal immune responses, and gut microbiota composition, potentially influencing intestinal inflammation [146,147,148]. This bidirectional interaction could at least explain to some extent the frequent occurrence of anxiety and depressive symptoms in patients with IBD, which are associated with impaired quality of life, increased symptom burden, and potentially less favorable disease outcomes [149,150,151]. For example, in a small experimental study in patients with IBD, FMT was associated with reduced depression, anxiety, and obsessive symptoms; however, it remains unclear whether these changes reflected direct microbiota–brain effects, improvement of gastrointestinal symptoms, or both [152]. Therefore, manipulation of gut microbiota could be considered an investigational strategy for neuropsychiatric outcomes in IBD rather than a treatment for depression or anxiety.

Collectively, the gut–brain axis extends the role of the microbiota in IBD beyond epithelial and immune homeostasis by integrating microbial metabolic activity, neuroimmune signaling, enteric nervous system function, and psychological health. Future longitudinal studies combining microbiome profiling with metabolomics, inflammatory biomarkers, disease-activity measures, and validated neuropsychiatric assessments are required to define causal pathways and identify patients who may benefit from microbiota-targeted interventions, which will be discussed in the upcoming sections in more detail.

## 3. Microbiota-Based Treatment Approach in IBD

The recognition that gut dysbiosis contributes to IBD pathogenesis has stimulated the development of microbiota-based therapeutic strategies. These approaches aim to restore microbial balance, improve epithelial barrier function, and reduce intestinal inflammation. Among them, FMT represents one of the most direct methods for re-establishing a healthy microbial ecosystem. In addition to FMT, other interventions such as probiotics, live biotherapeutic products, and genetically engineered microbes are also being explored for their therapeutic potential.

### 3.1. Fecal Microbiota Transplantation

FMT is a microbiota-based therapeutic approach aimed at restoring intestinal microbial balance by introducing a diverse and healthy donor-derived microbial community into the gastrointestinal tract [153,154,155]. The history of FMT is rooted in the 4th century in China, where stool-based extraction from a healthy individual was used to treat food poisoning and diarrhea [156,157], while its modern clinical use was first documented in 1958 in patients with pseudomembranous colitis [158]. Currently, FMT is used as treatment of recurrent *Clostridioides difficile* infection (CDI) with positive long-term outcomes [159]. Its high efficacy [30,159,160] is mediated through restoration of microbial competition [161], bile acid metabolism [162], and immunomodulation leading to subsequent improvement of gut barrier function [163]. CDI is particularly relevant for IBD since it disproportionately affects IBD patients [164]. The promising results in CDI have provided early proof-of-concept for FMT as a microbiome-correcting strategy for IBD [165] and is being actively explored as a therapeutic intervention for other gastrointestinal and extraintestinal conditions such as irritable bowel syndrome and type-1 diabetes mellitus, hypertension, and depressive disorders [166,167,168,169,170].

The translation of FMT into IBD therapy has been most extensively evaluated in UC and CD, where clinical trials have begun to characterize its efficacy and limitations. Clinical data in UC have shown that donor-derived FMT can induce remission and endoscopic improvement more effectively than placebo or autologous stool in a subset of patients [171,172,173,174]. In particular, pooled donor FMT resulted in significantly higher remission rates compared with autologous transplantation [173], and anaerobically prepared FMT was superior to placebo in achieving clinical remission [175]. Furthermore, the combination of FMT with an anti-inflammatory diet may help create a favorable niche for donor microbiota engraftment and sustain remission [176]. Similar to UC, in CD studies, positive clinical effects of FMT have been reported. In the first randomized sham-controlled pilot trial in CD, a single FMT did not meet the predefined donor-colonization endpoint at week 6, but it was associated with improvement in some clinical and inflammatory measures, including Crohn’s disease endoscopic index of severity (CDEIS) and C-reactive protein (CRP), compared with sham transplantation [177]. Another study has reported the improvement of several clinicopathological characteristics, including abdominal pain, hematochezia, and fever, as a response to FMT in CD patients [178]. A recent investigation in pediatric CD patients also reported the positive impact of encapsulated microbiota transplantation, with an increase in beneficial SCFA-producing bacterial genera and favorable modulation of immune response, by stimulation of anti-inflammatory cytokines [179]. Changes to microbial background can also be seen in CD patients with bile acid malabsorption, where FMT could alter bile acid-related metabolites and restore the metabolic balance [180]. However, larger trials in CD settings also demonstrated modest symptomatic relief, lack or absence of clinical/endoscopic remission, and room for side effects [178,181].

The positive outcome in IBD likely was widely attributed to the expansion of microbial diversity [182], reprogramming of the metabolic output, changes in microbial transcriptional activities [183], and an increase in regulatory T cells with parallel decrease in TH17 and CD8+ T cells [184].

Yet, despite these promising findings, therapeutic responses to FMT in IBD remain variable, and sustained remission has not been consistently demonstrated across all trials as systematically analyzed by Ikram and colleagues (2026) [185]. This heterogeneity likely reflects differences in donor microbial composition [186], recipient disease phenotype [187], treatment delivery approach and schedule, and accompanying treatments [188]. Therefore, donor selection and screening could be critical components of the procedure. Suitable donors are typically healthy adults without significant medical conditions or recent exposure to antibiotics, antivirals, antifungals, or probiotics, and donated stool must undergo extensive laboratory testing to minimize the risk of infectious transmission and other adverse events [189,190]. Recipient preparation, including recent medication review and timing of antibiotic discontinuation, as well as decisions regarding fresh versus frozen material and route of administration, may also influence treatment outcomes [191]. Overall, FMT represents one of the most direct strategies for microbiota modulation in IBD. As its clinical application remains constrained by inconsistent efficacy and unresolved questions regarding optimal donor and protocol selection, FMT is best viewed as a promising, but still incompletely standardized therapeutic approach in IBD.

### 3.2. Probiotics

Probiotics are defined as “live microorganisms that, when administered in adequate amounts, confer a health benefit on the host” [192]. These microorganisms are commonly delivered through food products and dietary supplements and include *Lactobacilli*, *Enterococci*, *Bifidobacteria*, and yeast species *Saccharomyces boulardii* [193], *Streptococcus* [194], *Clostridium*, and *Bacillus* [195]. Their effects on gut health depend on the specific strain and involve the production of antibacterial substances, including lactic acid, hydrogen peroxide, and bacteriocins [196,197,198]. Additionally, probiotics prevent the attachment of harmful microorganisms to epithelial cells [199], support epithelial integrity [200], modulate pH [201], and compete with pathogens for available nutrients [202]. Beyond these mechanistic actions, several practical advantages make probiotics particularly appealing for IBD research, including their wide presence in food, large variety of available strains, ease of administration [203], and potential for strain-level optimization through genetic engineering [204]. However, despite extensive investigation in murine IBD models, the gut microbiome of mice differs considerably from that of humans [205]. Therefore, this review focuses on clinical evidence from probiotic studies conducted in human IBD patients. As the microbiome of UC and CD patients varies [97], it is highly likely that different probiotics will be successful in the treatment of the two different forms of IBD.

#### 3.2.1. Probiotics in Treatment of Ulcerative Colitis

Recent evidence indicates that probiotic bacteria and yeast can attenuate intestinal inflammation in UC, as demonstrated in both preclinical models and clinical studies. Strains such as *Cyberlindnera jadinii* [206], *Kluyveromyces lactis* [206], *Lacticaseibacillus casei* [207], and *Bifidobacterium longum* [208] have been associated with restoration of microbial homeostasis, increased microbiome diversity, and reduced inflammation. However, the therapeutic efficacy of probiotics remains highly strain-specific and context-dependent, reflecting differential interactions with the host immune system and resident microbiota [209,210,211].

Despite encouraging mechanistic insights, single-strain probiotic interventions generally demonstrate modest and inconsistent efficacy in clinical settings, particularly when compared to multistrain formulations or adjunctive use with standard therapies. *Escherichia coli* Nissle 1917 (EcN) is among the most extensively studied single-strain probiotics in UC. Early trials suggested efficacy comparable to mesalazine in maintaining remission, with similar safety profiles [212,213]. However, subsequent studies reported conflicting outcomes, including lower remission rates in EcN-treated groups compared to placebo [214], underscoring variability in clinical response.

Similarly, *Lacticaseibacillus casei* has yielded heterogeneous results. While some clinical studies reported no significant differences in remission or relapse rates compared to placebo [215], others demonstrated immunomodulatory effects, including increased mucosal IL-10 and reduced expression of TLR4 and IL-1β [216]. Other single-strain candidates show more consistent benefits. For example, *Bifidobacterium longum* has been associated with improvements in clinical and endoscopic outcomes, as well as reduced UC activity indices [217]. *Lactobacillus rhamnosus* GG has demonstrated efficacy comparable to mesalazine in maintaining remission, with prolonged relapse-free intervals despite similar overall relapse rates, suggesting enhanced mucosal persistence or colonization capacity [218].

In contrast, multistrain probiotic formulations appear to exert more robust and reproducible anti-inflammatory effects. VSL#3, a composite formulation containing multiple *Lactobacillus*, *Bifidobacterium*, and *Streptococcus* species, has demonstrated clinical response and remission rates of up to 77%, along with consistent reductions in rectal bleeding and disease activity indices across several studies [219,220,221]. Similarly, the BIO-THREE formulation has shown efficacy in both active and quiescent UC, improving clinical and endoscopic parameters in refractory disease and maintaining remission in inactive cases [195,222]. BIFICO (Bifidobacterium triple viable capsule) has also been associated with reduced relapse rates in patients in remission, accompanied by increased abundance of species that predominate in a healthy microbiome and suppression of mucosal NF-κB activity [223].

Adjunctive use of probiotics alongside conventional pharmacological therapies further enhances therapeutic outcomes. Combination strategies with agents such as mesalazine or sulfasalazine consistently demonstrate greater reductions in disease activity and inflammatory markers compared to monotherapy. Better control of IBD symptoms has been achieved by combining different conventional therapies with supplementation of different bacteria. Examples are supplementing aminosalicylic acid (5-ASA) with VSL#3 [224], *Lactobacillus delbrueckii* and *Lactobacillus fermentum* [225] or EcN [226]. Similarly, the addition of BIFICO, a product consisting of bacterial strains *Bifidobacterium longum*, *Lactobacillus acidophilus*, and *Enterococcus faecalis*, to mesalazine-based regimens improved mucosal protection and reduced inflammatory activity [227]. Long-term studies further support these findings; a two-year trial demonstrated that supplementation with a probiotic blend (*L. salivarius*, *L. acidophilus*, *B. bifidum BGN4*) alongside mesalazine resulted in sustained clinical benefits, suggesting a role in maintaining long-term disease stability [228].

#### 3.2.2. Probiotics in Treatment of Crohn’s Disease

Unlike UC, CD can spread across the whole gastrointestinal tract (GIT), potentially lowering the effect of the microbiome. Up to now, most randomized studies failed to demonstrate significant superiority over placebo, including trials of *Lactobacillus rhamnosus* GG, *Lactobacillus johnsonii* LA1, *Saccharomyces boulardii*, and several multi-strain cocktails [229,230,231,232,233]. In particular, relapse prevention and postoperative recurrence were generally unaffected [234]. Only isolated findings suggested improvement, such as when *Saccharomyces boulardii* was combined with mesalazine, where relapse rates were reduced compared to standard therapy alone [235]. One pilot study consisting only of four patients showed that *Lactobacillus* GG alone can be helpful in some cases of CD [236].

As in the case of UC, a combination of different microorganisms results in a better result in the treatment of CD compared to individual bacteria or yeast. VSL#3 composition demonstrated similar results in the treatment of CD as in UC [237].

A mixture of probiotics mainly comprised *Bifidobacterium* and *Lactobacillus* together with prebiotics ameliorated symptoms in CD patients [238].

Another probiotic formulation combined with the enzyme amylase was associated with increased microbial diversity and a favorable shift in gut composition, characterized by enrichment of species prevalent under normal physiological conditions, such as *Lachnoclostridium* and *Mucispirillum schaedleri,* and reduction in pro-inflammatory bacteria including *Selenomonas lacticifex* in CD patients. Treatment also increased short-chain fatty acid production, supporting epithelial function and immune regulation, and was accompanied by changes in gene expression related to B cell activity and chemokine signaling. The presence of amylase was essential, as biofilm disruption enabled effective probiotic colonization, whereas these effects were absent without the enzyme [239]. Overall, more trials on much larger cohorts are needed to analyze the therapeutic efficacy of the microbiome in the treatment of CD. The summary of probiotic studies in clinical IBD research is provided in Table 1.

#### 3.2.3. Mechanisms of Probiotic Function

**Figure 2:**
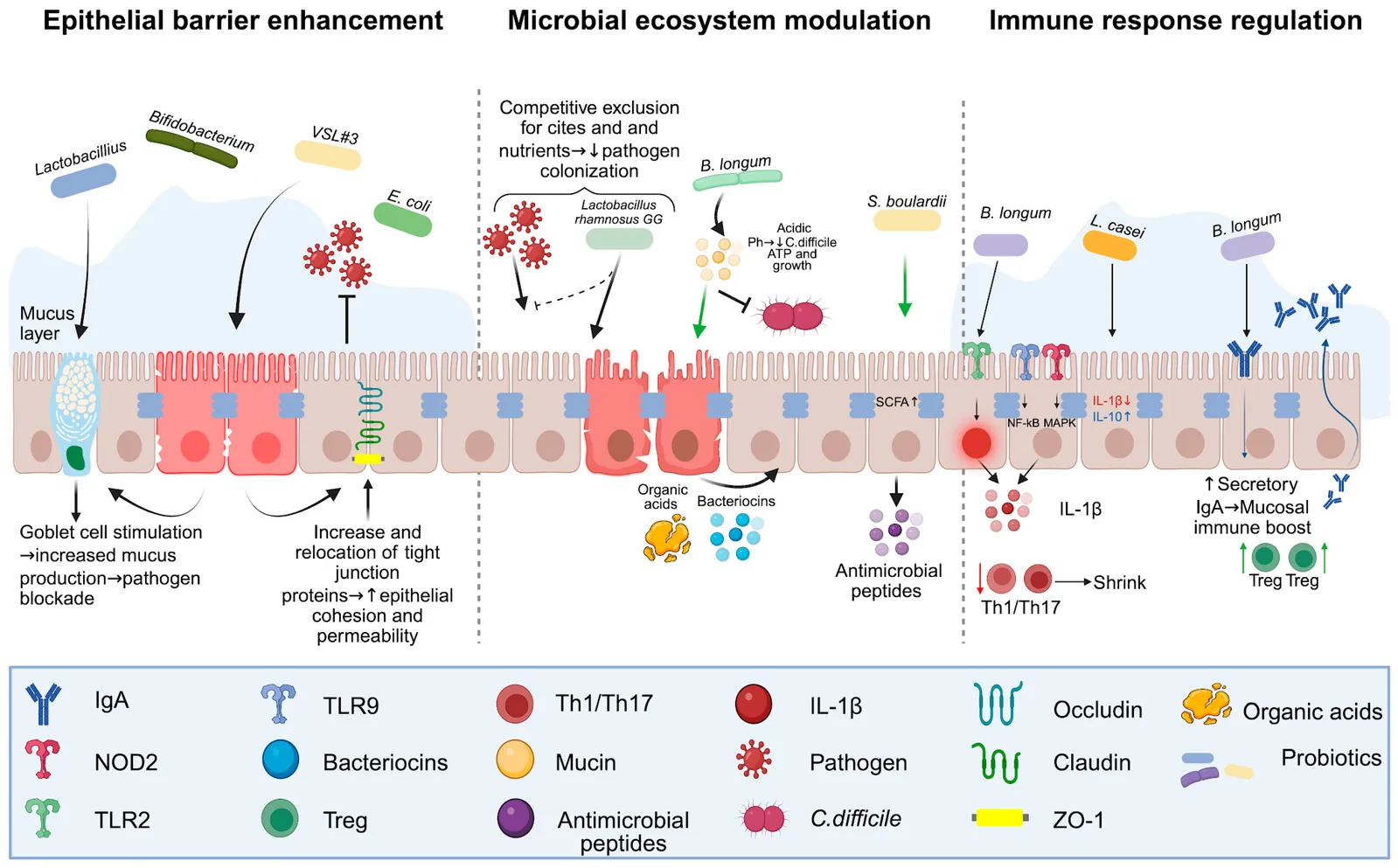
Mechanisms of probiotic therapeutic function. Epithelial Barrier Enhancement: Probiotics strengthen intestinal barrier function by stimulating mucin production, reinforcing tight-junction organization, and reducing epithelial permeability, thereby improving protection against luminal antigens and pathogens. Microbial Ecosystem Modulation: Inhibition of pathogen colonization through competitive exclusion and antimicrobial metabolite production while promoting microbial populations associated with a healthy microbiota, such as SCFA-producing bacteria and microbial diversity. Immune Response Regulation: Modulation of immune signaling pathways, regulation of cytokine production, promotion of epithelial repair, enhancement of mucosal IgA responses, and support of a shift toward immune homeostasis. Created in BioRender. Riethmacher, E. (2026), https://BioRender.com/0853mvb (accessed on 10 July 2026). Arrows: Black arrow—causal effect; green arrow—increase of respective component; red arrow—decrease of respective component; blunt-ended arrow—inhibitory effect, blue arrow—IgA expression path.

Although there is no clear classification of probiotics’ mechanisms of action, they are generally divided based on the process by which each group applies its positive effect within the intestinal environment. Such mechanisms include regulation of immune responses, modulation of the microbial ecosystem, and enhancement of epithelial barrier function (Figure 2) [245].

Acting as immediate or probiotic-mediated biological effectors, one of their key roles is to strengthen the epithelial barrier, responsible for the preservation against pathogens and luminal antigens. By stimulating goblet cells to produce mucins and upregulating tight-junction proteins such as claudin-1, probiotics enhance epithelial cohesion and reduce permeability [246]. An example of this can be seen with Lactobacillus plantarum, which raises occludin and ZO-1 levels and strengthens barrier function in cell models and in humans. The effect is associated with altered localization of these proteins within tight junctions. Additionally, increased expression levels of ZO-1 have been found after *Lactobacillus plantarum* treatment [200,247].

Probiotics can also compete with pathogens for adhesion sites and nutrients, thereby eliminating them from the intestine, a process known as competitive exclusion [202]. *Lactobacillus rhamnosus* GG adheres to intestinal epithelial surfaces via SpaCBA pili, with the SpaC subunit mediating binding to mucus and epithelial cells [248], thereby reducing adhesion of enteropathogenic *Escherichia coli* (EPEC) [249].

Many strains also produce antimicrobial substances such as bacteriocins, hydrogen peroxide, and organic acids, which inhibit pathogenic bacteria. *Bifidobacterium longum* produces organic acids such as lactate that can limit the growth of *C. difficile* by altering its metabolic activity. This includes lowering intracellular ATP levels, indicating that the inhibitory effect is mediated by acidic conditions [250]. Aforementioned EcN has been shown to compete directly with pathogenic bacteria, including *Salmonella*, *Shigella*, and invasive *E. coli* strains, by producing antimicrobial substances, reducing their adhesion and invasion of the gut lining, and limiting their presence within the colonic mucosa [251,252]. The multi-strain probiotic VSL#3 was shown to upregulate MUC gene expression [253], pro-IL-10 immune modulation and inflammatory cytokine suppression [254], leading to an enhanced microbial diversity, thereby suppressing pathogenic strains. Probiotics can also influence the metabolic environment in the gut, thereby positively influencing dysbiosis in IBD. The yeast Saccharomyces boulardii exerts its influence by increasing SCFA levels, particularly butyrate, providing an environment that supports SCFA-producing bacteria typically diminished in UC [255].

Beyond structural and ecological effects, probiotics are also able to regulate the innate and adaptive immune responses by secreting different molecules. These molecules interact with pattern-recognition receptors including TLR2, TLR9, and NOD2; they influence pathways such as NF-κB and MAPKs that govern cytokine production and epithelial renewal [256,257,258]. Bifidobacterium breve activates TLR2 signaling to promote epithelial repair while limiting inflammatory responses [259], and *Lacticaseibacillus casei* has shown beneficial properties via downregulation of pro-inflammatory mediators such as TLR-4 and IL-1β and upregulation of anti-inflammatory cytokine IL-10 within the mucosal tissue, which prevented relapses in UC patients and supported overall microbiota stability [259,260,261].

Probiotics have also been shown to increase anti-inflammatory regulatory T cells of the adaptive immune system, while reducing pro-inflammatory Th1 and Th17 cells [262]. Different strains of *Lactobacillus fermentum* have been shown to modulate Immunoglobulin A production individually and dependently on location in the gut [263]. *Bifidobacterium longum* also has been shown to enhance mucosal immunity by elevating secretory IgA and altering T-cell–related responses, including dampening Th1-associated cytokines and adjusting the Th17/Treg balance [264].

Together, these mechanisms support the stability of healthy gut microbiota, thereby increasing remission in UC models.

### 3.3. Live Biotherapeutic Products

FDA provides the definition for live biotherapeutic products (LBPs) as biological products that contain live organisms and are intended for the prevention, treatment, or cure of a disease [265]. Although some LBPs resemble probiotics in that they consist of beneficial live microorganisms, they differ in their intended use, as they are developed specifically to prevent or treat disease in patients rather than to promote general health. Thus, unlike probiotics marketed as dietary supplements for general health support, LBPs are regulated as medicinal products and face additional challenges related to microbial viability, batch consistency, and patient-specific microbiome variability [265]. This, in turn, should make them more reliable and consistent in the treatment of IBD compared to FMT or probiotics. Generally, these microbiome-based therapies work by rebuilding a healthy microbial ecosystem, reshaping metabolite production, and limiting pathogen overgrowth through colonization resistance [266,267].

Currently, no LBPs are available as standard treatment of IBD. However, there are a few LBPs at different stages of analysis.

BioPersist (SBT001), which is genetically engineered from the probiotic strain *Escherichia coli* Nissle 1917, modified to enhance its persistence in the inflamed intestine, and BioColoniz (SBT002), which is engineered from *Lactobacillus reuteri* and was modified to enhance intestinal colonization, have been analyzed in mouse and pig models. In the DSS colitis model, BioPersist produced broader anti-inflammatory and metabolic effects than its parent EcN. BioColoniz showed minimal metabolic differences compared with its parent *Lactobacillus reuteri* [268].

VE303, which contains eight clonal human commensal bacterial strains, has been tested positively in a mouse model to prevent infection by *C. difficile*, a serious side-effect in IBD, which exacerbates the symptoms of IBD. It has also successfully passed the Phase 1 clinical trial, showing no adverse effects in healthy humans [269].

VE202, which consists of strains derived from the human indigenous microbiota, has been studied in UC, but has not shown any significant advantage over placebo [242,270].

SER-287, composed of Bacillota spores, was also evaluated in patients with active mild-to-moderate UC. Although showing promising results in the Phase 1b trial, its Phase 2 trial was ultimately discontinued due to insufficient clinical benefit [243,271].

MB310, containing a defined consortium of eight live gut commensal bacterial strains, is currently being tested in a Phase 1b clinical trial and seems to have a positive effect on clinical remission [272].

RBX2660 contains a broad consortium of live microbes prepared, including members of Bacteroidota and Bacillota, and is also designated to restore microbial balance in patients with recurrent *C. difficile* infection. This LBP is already in Phase 3 of clinical trials and has shown good results as a treatment to reduce recurrent *C. difficile* infection [273,274]. So far, no trials on IBD patients without *C. difficile* infection have been conducted.

While LBPs propose a contemporary approach to IBD treatment, where formulations can be fine-tuned depending on case-specific microbial background, they have not yet been adopted in the general treatment of IBD. This proves the need for further research and the exploration of more effective combinations.

### 3.4. Genetically Engineered Microbiome

A particularly promising approach in microbiome-based IBD therapy involves the genetic engineering of safety-validated microbial strains to produce and deliver therapeutic molecules directly at the intestinal mucosa, which is the primary site of disease. This strategy has several advantages. To illustrate, genetically engineered microbiota can be administered orally on a daily basis, while the reactive compound does not have to pass through the acidic environment of the stomach, but rather is expressed in situ. Depending on the type of microbiome used, timely and local colonization can be achieved. Additionally, genetic engineering of microbiomes can be cost-effective.

Over the last 30 years, genetically engineered microbiomes have been analyzed as potential treatment options for IBD. However, up to now, no promising treatment for IBD using a genetically modified microbiome has been implemented in clinics. The main problems are the survival and colonization of these microbiomes in the gut as well as biosafety considerations.

To enhance the survival and colonization of the orally delivered microbiome, encapsulation, surface modifications, and targeting enhancements are now being used [275,276,277,278].

The risk of HGT is the most severe biological risk when using a genetically modified microbiome. This can be reduced by chromosomal integration of the gene rather than using plasmids [279].

The genes inserted in the genome of microbiota usually either reduce inflammation, increase epithelial integrity in the gut, or influence intestinal flora structure. The main microbial vehicles used so far are the bacterial strains *E. coli* Nissle 1917 [280,281,282,283,284,285] and *Lactococcus lactis* [286,287,288,289], as well as the yeast *Saccharomyces cerevisiae* [290,291]. Researchers have engineered the microbiome to reduce inflammation of the intestine by expressing and secreting anti-inflammatory cytokines such as IL-10 or, in low concentrations, IL-2 [244,282] or regulate inflammation via the expression of elafin (the inhibitor of neutrophil elastase) [281], heme oxygenase [286] or *schistosoma* immunoregulatory protein (Sj16) [285]. All of these approaches ameliorated the symptoms of IBD in a DSS-induced IBD mouse model. Only one analysis so far was done in human IBD patients [244].

Another approach was the expression of anti-IL-6-affibodies or TNF-a binding activity on the bacterial surface to capture the Il-6 in the surrounding environment, thereby also reducing inflammation [287,288]. It has also been shown that using a combination of these factors can lead to a better outcome [283].

To increase epithelial integrity, researchers have genetically modified bacteria to express catalase and superoxide dismutase to reduce ROS levels [289] or 5-Hydroxyindolacetic acid (5-HIAA), leading to an upregulation of tight-junction proteins [284].

**Figure 3:**
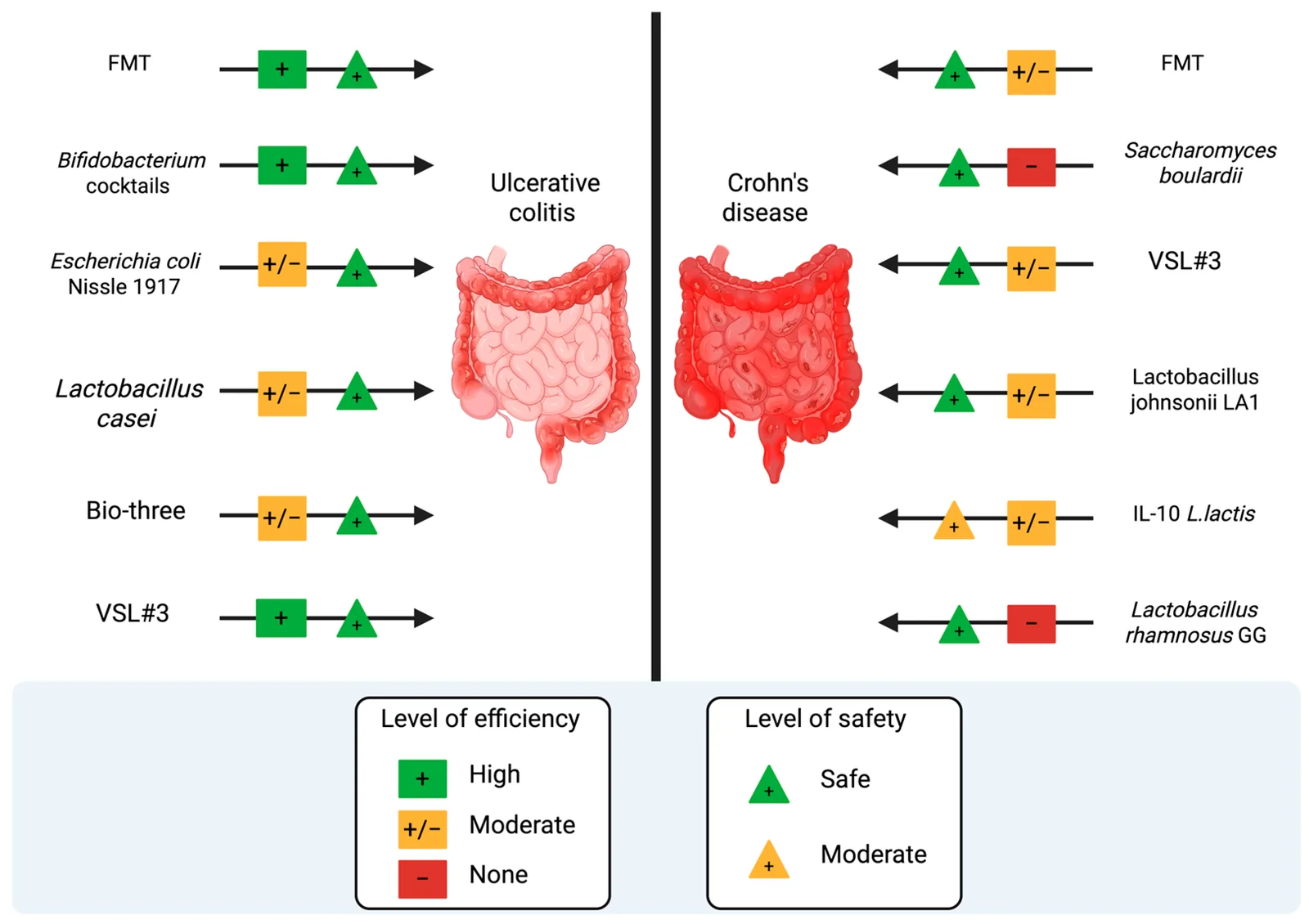
Schematic summary of the reported efficacy and safety of microbiome-based therapeutic approaches in ulcerative colitis and Crohn’s disease. The left panel summarizes therapeutic approaches evaluated in UC, and the right panel summarizes those assessed in CD. The interventions include fecal FMT and selected probiotic formulations, namely *Bifidobacterium* cocktails, *Escherichia coli* Nissle 1917, *Lactobacillus casei*, Bio-three VSL#3, *Saccharomyces boulardii*, *Lactobacillus johnsonii* LA1, IL-10-expressing *Lactococcus lactis*, and *Lactobacillus rhamnosus* GG. Squares indicate the reported level of efficacy (green, high; yellow, moderate; red, no demonstrated efficacy), and triangles indicate the reported level of safety (green, safe; yellow, moderate safety profile). Created in BioRender. Riethmacher, E. (2026) https://BioRender.com/edzor29 (accessed on 23 July 2026).

As it has been shown that dysbiosis is one of the main features in IBD, researchers have also successfully tried to influence the structure of the gut microbiome by genetically modified bacteria or yeast expressing either *schistosoma* immunoregulatory protein (Sj16) [285] or producing butyrate [291]. Also, levels of 5-HIAA have been shown to influence the structure of the gut microbiome [284]. A comparative summary of microbiota-targeted interventions is shown in Figure 3.

A more invasive treatment was conducted by Spisni et al. in 2015 [292]. They used nonpathogenic-invasive *Escherichia coli* as a vehicle for anti-COX2 siRNA. They showed that these bacteria are able to invade colon cells and reduce COX2 expression in an IBD mouse model, thereby ameliorating IBD symptoms [292].

Although all of the above-mentioned studies were successful in reducing inflammation of IBD, in the next step scientists are trying to specifically target genetically modified bacteria/yeast to the inflamed parts of the gut. Different approaches have been carried out to develop such a responsive delivery system. Extracellular Adenosine Triphosphate (eATP) is increased in inflammation. Based on this observation, *Saccharomyces cerevisiae* was modified to express a purinergic receptor with high eATP sensitivity. Activation of this receptor leads to the secretion of the ATP-degrading enzyme apyrase. This genetically modified yeast reduced inflammation in an IBD mouse model [290].

Making use of the increased nitric oxide (NO) levels in inflammation, *E. coli* Nissle 1917 has been modified to produce and secrete interferon-1, significantly reducing inflammation [280]. Another marker of IBD is a reduced level of SCFA such as butyrate and propionate. Researchers used this to generate a biosensor made up of *E. coli* able to express the inserted gene in the presence of low amounts of SCFA [293].

As most studies up to now were done in mouse models of IBD, not much is known yet about their efficacy in IBD patients. Several issues have to be solved before genetically modified microbes can be used in the treatment of IBD. The most important issue is biosafety. It has to be ensured that genetically modified bacteria are contained in the patient and no HGT will occur. With new methods using chromosomal integration instead of plasmids, HGT is highly unlikely. To contain the bacteria/yeast used, it might be advantageous to use Generally Recognized as Safe (GRAS) strains not usually colonizing the human gut. This will ensure that the genetically modified strains will not spread among the population. Although this is in contrast to many publications stating it would be important to use strains with high colonization ability in the gut [294], it would be good to take it into account when developing new treatments using genetically modified microbiota.

## 4. Conclusions

Current evidence supports the gut microbiota as an important contributor to the pathogenesis of IBD [35,36,37,38,39] and a plausible target for therapeutic intervention. Dysbiosis in IBD is characterized not only by reduced microbial diversity and depletion of species typically abundant in the gut under normal health conditions but also by expansion of pathobionts and broader functional disturbances that may sustain mucosal inflammation [33,99,103]. These observations have prompted the development of microbiota-directed strategies aimed at restoring microbial balance and improving disease control.

Microbiota-based approaches, including FMT, probiotics, live biotherapeutic products, and genetically engineered microbes, have shown promising but variable results. Among them, FMT currently provides the strongest proof-of-concept for microbiome restoration, particularly in UC [173], whereas probiotic efficacy remains strain-specific and generally more limited in CD [178,181]. At the same time, newer defined microbial consortia and engineered bacterial platforms offer the advantage of better standardization [265], mechanistic precision, and the potential for targeted delivery of anti-inflammatory or barrier-protective functions [275,282,284]. However, challenges related to donor selection [153], engraftment, durability of response, safety, regulatory approval [185,189], and interindividual variability continue to restrict broad clinical translation [269].

Overall, microbiota-based therapies remain promising, but they are not yet ready to replace established treatment strategies in routine IBD care. Their future role will likely depend on improved patient stratification, more rigorous mechanistic understanding, standardized treatment protocols, and better integration of microbial, immunological, and clinical data. Additionally, as current evidence is largely derived from multistrain formulations, the role of individual probiotic strains remains insufficiently characterized. Focusing on single-strain solutions may clarify their specific functions and reveal benefits in strains that have yet to be investigated. A clearer understanding of host–microbiome interactions may ultimately help translate microbiome research into safer, more effective, and more personalized therapeutic options for patients with IBD.

## Figures and Tables

**Table 1:** Summary of probiotic studies in clinical IBD research.

Aims	Sampling Population	Type of IBD	Yeast/Bacteria Strain	REM	REL	ACT	END	INF	BAR	MIC	COL	Reference
To compare oral *Escherichia coli* Nissle 1917 with mesalazine for maintenance of remission in UC	Adults 18–70 y, UC in remission, n = 327	UC	*Escherichia coli* Nissle 1917	n/a	=	0	0	·	·	·	·	[240]
To compare a non-pathogenic *Escherichia coli* preparation with mesalazine for maintenance therapy	Adults, active UC n = 116	UC	Non-pathogenic *Escherichia coli* Nissle 1917	0	+	·	·	·	·	·	·	[213]
To evaluate the effects of non-pathogenic *Escherichia coli* In UC settings	Adults, quiescent UC n = 120	UC	Non-pathogenic *Escherichia coli*	n/a	0	0	·	·	·	·	·	[212]
To test clinical efficacy of VSL#3 in patients with active UC	Adults, mild–moderate active UC n = 24	UC	VSL#3 mixture (*Lactobacillus casei*, *Lactobacillus plantarum*, *Lactobacillus acidophilus*, and *Lactobacillus delbrueckii* subspecies *Bulgaricus*)	(+)	·	(+)	(+)	0	·	·	·	[220]
To assess the supplementary role of VSL#3 in patients with UC experiencing relapse under 5-ASA treatment	Adults, relapsing mild–moderate UC on stable 5-ASA ± immunosuppressant n = 144	UC	VSL#3 mixture (*Lactobacillus casei*, *Lactobacillus plantarum*, *Lactobacillus acidophilus*, and *Lactobacillus delbrueckii* subspecies *Bulgaricus*) + 5-ASA	+	·	++	++	·	·	·	·	[224]
To reevaluate effects of *Escherichia coli* Nissle 1917 as add-on therapy in UC patients	Adults, mild–moderate UC n = 118	UC	*Escherichia coli* Nissle 1917 (Mutaflor) + 5-ASA	0	n/a	0	++	·	·	0	·	[226]
To test ciprofloxacin and/or orally administered *Escherichia coli* Nissle 1917 as add-on to conventional therapy in active UC	Adults, active UC n = 100	UC	*Escherichia coli* Nissle 1917	−−	·	−−	·	·	·	·	·	[214]
To assess the effects of *Lactobacillus casei* strain ATCC PTA-3945 in treating ulcerative colitis	Adolescents and adults, relapsing or newly diagnosed UC n = 34	UC	*Lactobacillus casei* strain ATCC PTA-3945	0	0	·	·	·	·	·	·	[215]
To evaluate rectal administration of *Lactobacillus casei* on colonic microbiota, mucosal cytokine balance, and toll-like receptor expression	Adults, mild left-sided UC n = 26	UC	*Lactobacillus casei* DG	·	·	0	·	++	·	++	·	[216]
Investigate induction of remission in the Japanese population with UC	Adults, mild–moderate active UC n = 56	UC	*Bifidobacterium longum* 536	+	·	(+)	(+)	·	·	·	·	[217]
To evaluate the treatment potential of *Lactobacillus rhamnosus* GG as adjunctive and standalone therapy in UC	Adults, quiescent UC n = 187	UC	*Lactobacillus rhamnosus* GG	n/a	0	·	·	·	·	·	·	[218]
Evaluate safety and efficacy of the VSL#3 formulation and its components	Adults, mild–moderate active UC n = 34	UC	VSL#3 mixture (*Lactobacillus casei*, *Lactobacillus plantarum*, *Lactobacillus acidophilus*, and *Lactobacillus delbrueckii* subspecies *Bulgaricus*)	(+)	·	(+)	·	·	·	·	·	[219]
Test remission induction potential of the VSL#3 probiotic formulation in the active UC population	Adults, mild–moderate active UC n = 147	UC	VSL#3 (*Lactobacillus paracasei*, *Lactobacillus plantarum*, *Lactobacillus acidophilus*, and *Lactobacillus delbrueckii* subspecies *bulgaricus*, *Bifidobacterium longum*, *Bifidobacterium breve*, and *Bifidobacterium infantis*, *Streptococcus thermophilus*)	++	·	++	++	·	·	·	·	[221]
Evaluate efficacy of probiotics compared to conventional therapy.	Adults, mild–moderate distal UC refractory to or intolerant of conventional therapy n = 20	UC	*Streptococcus faecalis T-110*, *Clostridium butyricum TO-A*, *Bacillus mesentericus TO-A*	(+)	·	(+)	(+)	·	·	(+)	·	[222]
Test relapse prevention potential of probiotics in inactive UC	Adolescents and adults, UC in remission (CAI ≤ 5) n = 60	UC	*Streptococcus faecalis T-110*, *Clostridium butyricum TO-A*, *Bacillus mesentericus TO-A*	0	+	·	·	·	·	0	·	[195]
Assess relapse prevention capacity of the BIFICO in UC patients	Adults, UC in remission after sulfasalazine + glucocorticoid n = 30	UC	BIFICO (*Enterococcus*, *Bifidobacterium*, *Lactobacillus*)	·	++	·	·	++	·	++	·	[223]
Evaluate combination therapy of mesalazine with Bifid Triple Viable Capsules	Adults, mild–moderate UC n = 360	UC	Mesalazine + Bifid Triple Viable Capsules (*Lactobacillus acidophilus*, *Bifidobacterium*, *Enterococcus*)	·	·	++	·	++	·	·	·	[227]
Test anti-inflammatory and therapeutic effects of combination of probiotics with conventional therapy	Adults, newly diagnosed mild–moderate UC n = 40	UC	*Lactobacillus delbrueckii*, *Lactobacillus fermentum* + sulfasalazine	·	·	·	·	++	·	·	·	[225]
Evaluate combination of mesalazine with multistrain probiotic formulation	Adults, moderate–severe UC n = 60	UC	Mesalazine + *Lactobacillus salivarius*, *Lactobacillus acidophilus*, *Bifidobacterium bifidum* BGN4	·	·	++	++	·	·	·	·	[228]
To evaluate the effectiveness of the merged probiotic formula in active CD patients	Adults, active CD with no post-operative history n = 10	CD	*Bifidobacterium breve*, *Bifidobacterium longum*, *Lactobacillus casei* + psyllium	(+)	·	(+)	·	·	·	·	·	[238]
Assess pediatric CD therapy in children with active CD	Children, mild–moderate active CD n = 4 enrolled	CD	*Lactobacillus rhamnosus* GG	·	·	(+)	·	·	(+)	·	·	[236]
Assess effects of the probiotic to maintain medically induced remission	Adults, mild–moderate active CD n = 11	CD	*Lactobacillus rhamnosus* GG	0	0	0	·	·	·	·	·	[229]
Test the capacity of a probiotic to enhance standard maintenance therapy remission in CD	Adults, CD in remission induced by steroids or salicylates n = 165	CD	*Saccharomyces boulardii*	n/a	0	0	·	0	·	·	·	[232]
To evaluate the effects of *Saccharomyces boulardii* as add-on therapy for relapse prevention	Adults, CD in clinical remission n = 32	CD	*Saccharomyces boulardii* + mesalamine	n/a	++	·	·	·	·	·	·	[235]
Test the effects of *Saccharomyces boulardii* on the intestinal permeability in patients with CD	Adults, CD in remission on baseline therapy n = 34	CD	*Saccharomyces boulardii* + baseline medication (mesalamine, azathioprine, prednisone, metronidazole and/ or thalidomide)	·	·	·	·	·	(+)	·	·	[231]
To test the prevention capacity of a probiotic in early postoperative recurrence of CD	Adults, CD after curative ileo-caecal resection n = 98	CD	*Lactobacillus johnsonii* LA1	n/a	0	·	+	·	·	·	·	[230]
To assess microbiological and immunological effects of synbiotic therapy in active CD	Adults, active CD n = 35	CD	*Bifidobacterium longum* + Synergy 1	·	·	(+)	·	(+)	·	(+)	·	[241]
Test efficacy of multistrain probiotic formulation for mitigation of inflammation and quality of life improvement in mild–moderate CD and UC patients	Adults, quiescent/mild UC n = 81	UC	*Lactobacillus acidophilus*, *Lactiplantibacillus plantarum*, *Lacticaseibacillus rhamnosus*, *Enterococcus faecium*	n/a	+	0	·	(+)	·	·	·	[233]
Test efficacy of multistrain probiotic formulation for mitigation of inflammation and quality of life improvement in mild–moderate CD and UC patients	Adults, quiescent/mild CD n = 62	CD	*Lactobacillus acidophilus*, *Lactiplantibacillus plantarum*, *Lacticaseibacillus rhamnosus*, *Enterococcus faecium*	n/a	0	0	·	0	·	·	·	[233]
To test whether VSL#3 prevents postoperative CD recurrence	Adults, CD within 30 d of ileocolonic resection n = 120	CD	*Lactobacillus acidophilus*, *Lactobacillus plantarum*, *Lactobacillus paracasei*, *Lactobacillus delbrueckii subsp. bulgaricus*, *Bifidobacterium breve*, *Bifidobacterium longum*, *Bifidobacterium infantis*, *Streptococcus salivarius* subspecies *thermophilus*	n/a	·	0	+	++	·	·	·	[237]
Evaluate safety and tolerability of multistrain probiotic consortium	Healthy adults, vancomycin pre-treated n = 31	UC	VE202: 12 strains of *Clostridium* cluster XIVa, 2 strains of cluster IV, and 2 strains of cluster XVIII	n/a	n/a	n/a	n/a	·	·	·	++	[242]
Evaluate safety and efficacy of SER-287 in UC patients	Adults, active mild–moderate UC n = 58	UC	SER-287: *Bacillota* spore-based microbiome therapeutic	+	·	0	+	0	·	++	++	[243]
To assess the safety and efficacy profile of modified, IL-10-expressing *Lactococcus lactis*	Adults, moderate–severe CD n = 10	CD	Genetically modified *Lactococcus lactis* secreting human interleukin-10 (LL-Thy12)	(+)	·	(+)	·	·	·	·	(+)	[244]

Endpoints: REM—clinical remission; REL—relapse; ACT—disease activity index; END—endoscopic activity; INF—inflammatory and immune markers; BAR—intestinal barrier permeability; MIC—microbiota and metabolome: taxa commonly found in healthy conditions, SCFA, bile acids, 16S/T-RFLP profiles; COL—engraftment, magnitude metrics: “++”—significant benefit (*p* < 0.05), “+”—numerical benefit, “(+)”—significant change from baseline in the treatment group only, difference between treatment groups is absent. “0”—no difference; “−−”—significantly worse with treatment; “=”—equivalence to the compared treatment; “·”—not given; “n/a”—not applicable to this design.

## Data Availability

No new data were created or analyzed in this study. Data sharing is not applicable to this article.

## Abbreviations

The following abbreviations are used in this manuscript:

IBD	Inflammatory Bowel Disease
IBDU	Inflammatory Bowel Disease Unclassified
CD	Crohn’s Disease
UC	Ulcerative Colitis
CDI	*Clostridioides difficile* Infection
GIT	Gastrointestinal Tract
CDEIS	Crohn’s Disease Endoscopic Index of Severity
CRP	C-Reactive Protein
5-ASA	5-Aminosalicylic Acid
FMT	Fecal Microbiota Transplantation
LBP	Live Biotherapeutic Products
EcN	*Escherichia coli* Nissle 1917
BIFICO	Bifidobacterium Triple Viable Capsule
EPEC	Enteropathogenic *Escherichia coli*
OMVs	Outer Membrane Vesicles
TLR-4	Toll-Like Receptor 4
NOD2	Nucleotide-Binding Oligomerization Domain-Containing Protein 2
MAM	Mucosa-Associated Microbiota
Th1	Type 1 Helper T Cells
IL-10	Interleukin 10
IgA	Immunoglobulin A
ZO-1	Zonula Occludens-1
SCFAs	Short-Chain Fatty Acids
SCCA	Simple Clinical Colitis Activity
HGT	Horizontal Gene Transfer
5-HIAA	5-Hydroxyindolacetic Acid
COX2	Cyclooxygenase-2
eATP	Extracellular Adenosine Triphosphate
NO	Nitric Oxide
GRAS	Generally Recognized as Safe
CAI	Colitis Activity Index
